# Peacebuilding in times of COVID-19: risk-adapted strategies of cooperation and development projects

**DOI:** 10.1007/s42597-020-00040-2

**Published:** 2020-11-04

**Authors:** Luca Eufemia, Camilo Lozano, Tatiana Rodriguez, Martha Del Rio, Héctor Morales-Muñoz, Michelle Bonatti, Stefan Sieber, Katharina Löhr

**Affiliations:** 1 Leibniz Centre for Agricultural Landscape Research (ZALF), Müncheberg, Germany; 2 Agricultural Sciences, Humboldt-Universität zu Berlin, Berlin, Germany; 3 Internacional Center for Tropical Agriculture (CIAT), Cali, Colombia; 4 Agricultural Economics, Humboldt-Universität zu Berlin, Berlin, Germany; 5 Urban Plant Ecophysiology, Humboldt-Universität zu Berlin, Berlin, Germany

**Keywords:** Pandemic, Conflict, Peacebuilding, Risk-adapted strategies, Cooperation, Pandemie, Konflikt, Friedensbildung, Risikoadaptierte Strategien, Kooperation

## Abstract

National and international cooperation and development projects (CDP) are fundamental for peacebuilding. However, unforeseen global crises, like COVID-19, can endanger such projects, requiring rapid adaption. In Colombia, the coronavirus outbreak threatens to slow the implementation of peace-related projects, while simultaneously violence over control and ownership of land increases. Although the mid- to long-term consequences for peacebuilding are unknown, exploring risk-adapted strategies of national and international CDP can help identify crucial aspects for future processes and implementations. This study explores the key challenges and coping strategies of implementing agencies and stakeholders to COVID-19, thus helping to derive and improve risk-adapted strategies. After reviewing academic and grey literature, and implementing a semi-structured survey, peacebuilding risked-adapted strategies to COVID-19 are explored with respect to conflict-affected and vulnerable areas of Colombia. Findings show that increasing complexity rooted in top down governmental measures, the rise of new local power relations (e.g. armed groups, illicit activities), and social alienation are negatively affecting peacebuilding in Colombia. Future CDP risk adapted strategies should build on local interests and needs through public-private and environmental cooperation.

## Abstract (de)

Nationale und internationale Kooperations- und Entwicklungsprojekte (CDP) sind für die Friedensförderung von grundlegender Bedeutung. Unvorhergesehene globale Krisen, wie COVID-19, können solche Projekte jedoch gefährden und erfordern eine rasche Anpassung. In Kolumbien droht der Ausbruch des Coronavirus die Durchführung friedensbezogener Projekte zu verlangsamen, während gleichzeitig die Gewalt über die Kontrolle und den Besitz von Land zunimmt. Obwohl die mittel- bis langfristigen Folgen für die Friedenskonsolidierung unbekannt sind, kann die Untersuchung risikoangepasster Strategien des nationalen und internationalen CDP dazu beitragen, entscheidende Aspekte für künftige Prozesse und Implementierungen zu ermitteln. Diese Studie untersucht die Herausforderungen und Bewältigungsstrategien der Durchführungsorganisationen und Interessengruppen im Kontext von COVID-19, umso risikoangepasste Strategien abzuleiten und zu verbessern. Basierend auf Literaturrecherche und der Durchführung semi-strukturierter Interviews werden risikoangepasste Strategien der Friedensförderung für COVID-19 in unterschiedlichen Teilen Kolumbiens untersucht. Die Ergebnisse zeigen, dass die zunehmende Komplexität, die in den Maßnahmen der Regierung von oben nach unten, dem Entstehen neuer lokaler Machtverhältnisse (z. B. bewaffnete Gruppen, illegale Aktivitäten) und in zunehmend sozialer Entfremdung wurzelt, die Friedensförderung in Kolumbien negativ beeinflusst. Künftige risikoangepasste Strategien des CDP sollten auf lokalen Interessen und Bedürfnissen durch öffentlich-private und umweltpolitische Zusammenarbeit aufbauen.

## Introduction

National and international cooperation and development projects (CDP) play a fundamental role in peacebuilding processes (Fischhendler and Tenenboim-Weinblatt 2019; Amego Korbla Penu and Wellington Essaw 2019; Lemon and Pinet 2018; Jaruma 2013). Managing CDPs, challenging under normal circumstances, requires agility and flexibility when confronting unforeseen global emergencies, like the outbreak of coronavirus (COVID-19). Without rapidly adapting management approaches, CDP might be endangered. UNDP (2020) notes the impact of COVID-19 is likely to be worse in conflict-affected countries, where past and present vulnerabilities in health and governance could be amplified, thus exacerbating violence. Further, seven of the ten most-vulnerable countries to pandemics are in conflict zones (Moore et al. 2017), places where cross-cutting and chronic dimensions of violence, like illicit markets, unemployment, economic disparities between regions, marginalisation, displacement, exploitation, conflicts over local issues, lack of access to public services (e.g. health, infrastructures), and corruption increase risks and the duration of conflicts (Sánchez-Zamora et al. 2017; Flores and Vargas 2018). Yet, health, humanitarian, and cooperation strategies should be stimulated simultaneously to sustain the most vulnerable during the pandemic—like displaced populations—to both the virus itself and those measures enacted to fight it (San Lau et al. 2020).

The forthcoming COVID-19 depression could result in funding cuts to peace operations, endangering their ability to achieve benchmarks, while hampering critical peacebuilding actions like the protection of civilians, protection of social leaders, financial support to ex-combats, and the protection of key infrastructure (Garzón et al. 2020). Reduced funding can also affect local organizations based on projects. Furthermore, the shrinking of peace related budgets limits CDP capacity to hire highly-qualified personal if not keep pre-COVID-19 personnel employed. CDP support to governments, institutions, and conflict parties is likely to be limited in the mid- to long-term (de Coning 2020; Dorussen 2020). At the administrative level, COVID-related constraints oblige peacebuilding CDP organizations to shift to an “essential” frame of mind, forcing a re-examination of activities and interventions, while identifying new threats developing alongside the pandemic and evolving governmental measures. This is challenging due to the multiple and changing faces of the COVID-19 pandemic. Among other aspects, coordinating activities is difficult due to poor internet access, low penetration of mobile networks, electricity cuts, and digital illiteracy in conflict-affected rural areas. In this context, additional funds are being requested to invest in online communication and information systems (Ansorg and Strasheim 2020).

In Colombia, the COVID-19 pandemic is worsening the underlying roots of conflicts, especially in rural contexts beset with unsecure land distribution, poor access to public services, and high levels of inequalities (Peace Direct 2020). In some places, physical and psychological violence is increasing, especially against social, indigenous and environmental leaders, former guerrillas and Venezuelan migrants (Sandoval et al. 2020). Besides, public health measures, including social distancing, lockdowns, and public virtual attendance, trigger delays, slowing the implementation of the 2016 peace agreement between Colombia’s government and the Revolutionary Armed Forces of Colombia (FARC). Two immediate consequences exist: First, scarce human and financial governmental capacities show that they are failing to control and monitor isolated and remote regions endowed with natural resources. This not only facilitates the expansion of the drug trafficking business, but is also multiplying practices of violence such as forced recruitment, displacement, land grabbing, and deforestation (Garzón et al. 2020). Second, the COVID-19 outbreak reveals emerging obstacles to the effective implementation of governmental peacebuilding strategies, such as the Programs of Development with a Territorial Focus or Programas de Desarrollo con Enfoque Territorial (PDET) or the Municipal and Departmental Development Plans (rural roads, productive and community infrastructure, schools, health facilities, etc.). The lack of funding, the low degrees of political and policy processes at the regional and local level, methodological inconsistencies in the design of participatory approaches and the increased violence against community leaders are preventing the effective implementations of these programs and their cooperation with CDPs.

Notwithstanding these developments, COVID-19 also provides opportunities to promote peace and to advance peacebuilding. Examples include the global ceasefire campaigns (United Nations Press 2020), the immediate impacts on environmental and climate change resulting from lockdowns (e.g. exceptional drops in carbon dioxide emissions) (Eufemia and Hussein 2020), community-based initiatives (e.g. indigenous COVID-19 prevention and containment, focusing on voluntary collective isolation and contact-tracing) (Kaplan et al. 2020), reorientation of ex-guerrilla combatants’ productive projects (e.g., from textile production to mask manufacturing), and the use of ICT in transitional justice processes (UN 2020). Although the veracity, impact, and transferability of these advances is unclear, the visible manifestation of the COVID-19 crisis in post conflict scenarios reflect increased complexities and changes in peacebuilding scenarios that also affect the planning and functioning of CDPs.

By investigating the key challenges and coping strategies of implementing agencies and stakeholders to COVID-19, the main aims of this work are to find (i) how CDPs adapt to perceived risks, central government management plans, and funding agency requirements; and (ii) which impact the pandemic has on peacebuilding and corresponding CDP responses. In the following sections, we present our methodology formed by the case study areas and the semi-structured interviews, the results visualizing the main findings, followed by the discussion that addresses key insights against the current situation of both the peacebuilding endeavour and the COVID-19 crisis. Finally, the conclusion summarizes the whole content and it offers suggestions to improved risk-adapted strategies.

## Methodology

### Case study areas

**Fig. 1:**
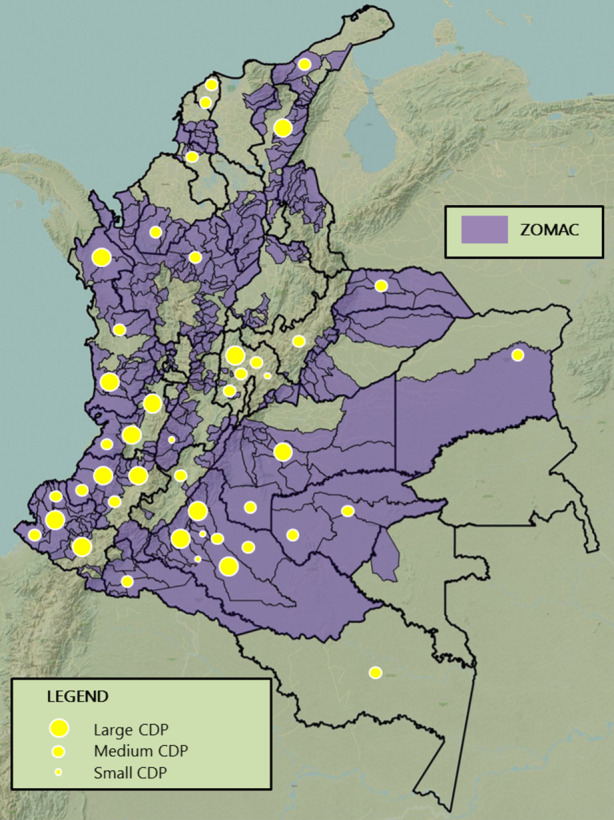
ZOMAC and applied surveys. (Source: Adapted from © ZOMAC, Sistema Informativo del Gobierno de la Republica de Colombia—SIG)

Since the 2016 peace agreement, Colombia’s peacebuilding process has experienced numerous and complex challenges and changes of power structures regarding land use and ownership; resulting risks include the strengthening of old and new armed groups (Graser et al. 2020). For this reason, our methodological take includes territoriality, as we develop a visualization of conflict-affect areas. We investigate the impact of the COVID-19 crisis on national and international CDPs, targeting projects in some of the most conflict-affected and vulnerable areas of Colombia. Fig. 1 overlays the “Areas Most Affected by Armed Conflict” (ZOMAC), as listed in governmental decree n. 1650 on October 9, 2017 (Republic of Colombia 2017), with the applied 10 surveys, addressing 13 CDPs implemented across multiple regions as shown by dots (a complete list is in the Appendix), including 7 large (with a budget of over 1 million €), 3 medium (with a budget less than 1 million € and implemented at a national scale), and 3 small (with a budget less than 1 million € and implemented at a local scale) projects.

### Semi-structured interviews

A list of running CDPs was obtained through internet and institutional networks. Emails and phone calls with key actors evaluated their willingness to participate. An interview protocol was developed to guide semi-structured interviews with project managers and stakeholders of Colombian CDPs. The questionnaire comprises four main blocks: (i) context: expertise (e.g. peace-related, national, and international) gender, and age; (ii) information about the reference CDP: scale (e.g. local, regional, or national), duration, approximate funding, project role, and project team; (iii) the perceived impact of COVID-19 on project implementation: immediate perception of issue extent, proximity, immediate responses, first effects, and perception regarding governmental responses at all levels (local, regional, and national), as well as of funding agencies and direct donors; and (iv) risk-adapted strategies: evaluation of existing strategies, adoption of new strategies and assets, and the future outlook on desirable risk-adapted strategies. Survey participation was voluntary and personal data kept anonymous.

While using qualitative methods to categorize each block, quantitative approaches were used for descriptive statistics to present numeric values. In total, 10 interviews were conducted, addressing the work of 13 CDPs, covering most ZOMAC. The interviews were conducted in Spanish via phone or skype, each lasting between forty-five minutes and two hours. Each interview was recorded for further data analysis.

## Results

Relevant findings concerning COVID-19 impacts, CDP responses, and risk-adapted strategies are collected in blocks (iii) and (iv) of the survey (see 2.2 Methods, Step 2. Survey). Tables 1 and 2 present the key interfaces and relations. Grey boxes indicate overlapping perceptions between large, medium, and small CDPs.

**Table 1:** COVID-19 impacts on CDP and CDP responses

Related question	CDP Large	CDP Medium	CDP Small
CDP evaluation before COVID-19 (on a scale from 1, very negative, to 5, very positive)	4.3	4.5	3.7
First perception of COVID-19	Conspiracy (e.g. social media)	–	–
	Surprise (e.g. unexpected event)		–
	–	Collective paranoia (e.g. health emergency, people at higher risk from COVID-19, social-distancing, and community-lockdown)	
	Distant (e.g. event happening elsewhere far away)	–	–
	Long-term changes (e.g. lifestyle etc.)	–	–
Impact of COVID-19 on CDP (on a scale from 1, none, to 5, substantial)	4.3	4	5
Internal adaptation components	CDP on hold, and evaluation of expected new planning (e.g. virtual meetings with directors and donors)	–	CDP on hold and evaluation of expected new planning (see CDP Large, this row)
	CDP converted (e.g. from physical activities to virtual platforms, radio, home office)		–
	Informal measures (e.g. redesign of power relations in remote areas and interaction with armed groups)	–	Informal measures (see CDP Large, this row)
	Control and monitoring system of entry-exit from and in local communities (e.g. wellbeing, health, analysis of increasing risks)	Limit to co-create activities in the field with local communities (e.g. social and educational processes.)	Increased reliance on subsistence agriculture for food (e.g. manioc, banana)
	Biosecurity programmes (e.g. diagnostic kits and agro ecological tools in remote project areas)		
	Funds relocation in accordance with donors (e.g. from training to campaigns)		
	Increased trust at the community level in the most remote places (e.g. solidarity in areas with no electricity nor access to health services)		
External adaptation components	Government measures (e.g. top down system with delegation functions to regional and local governments, increased online capacity building about local economic activities, public funds destined to the most vulnerable families)		
	Increased bureaucracy alongside government measures (e.g. lack of coordination at multiple scales)		–
	Increased (endemic) corruption (e.g. humanitarian aid involved)	–	Increased (endemic) corruption (see CDP Large, this row)
	Increased violence and threats against social/environmental leaders, human rights activists		
	Safe guards applied (e.g. guidelines from donors.)	Lack of transparency from central governments (e.g. communication was misleading and often COVID-19 data were not accurate)	Biosecurity protocol for local economic activities (e.g. official label to increase demand of local products and services such as eco-tourism)
	–	Increased food insecurity (e.g. in border areas that import most food from other countries)	–

Source: Authors’ own elaboration

**Table 2:** Risk adapted strategies of CDP

Related question	CDP Large	CDP Medium	CDP Small
Relevance of existing CDP risk-adapted strategies before COVID-19 (on a scale from 1 none, to 5 substantial)	2.7	3.2	1.3
Impact of COVID-19 on peacebuilding processes within CDP	Negative impacts on peoples’ perception of peacebuilding (e.g. increased territorial control of armed groups, restrictions of mobility in/out communities, less personal security)	–	Negative impacts on peoples’ perception of peacebuilding (see CDP Large, this row)
	–	Negative impacts due to misleading data communication and monitoring (e.g. lack of transparency, questioning data gathering in remote and disconnected areas, less institutional trusts)	–
	Learning opportunity for peacebuilding processes (e.g. the risks embedded in a centralized system, the perceived increase of institutional corruption, the interruption of most implementations of the peace deal with the FARC, the importance of human rights, gender equality)		–
	Reconsider schemes for territorial planning to foster environmental projects (e.g. sustainable development is the only way to achieve peace)	Increased violence and threats against social/environmental leaders, human rights activists	Increased inequalities (e.g. marginalization of remote places and communities)
	Risks of less funds for cooperation and peacebuilding in the future	Increased environmental deteriorations (e.g. less control equals more deforestation)	–
Suggested risk-adapted strategies to CDP implementation	Support private-public agreements to sustain small-scale and medium economic activities (e.g. mechanisms to improve governability at the local level)	–	Support private-public agreements to sustain small-scale and medium economic activities (see CDP Large, this row)
	To create new forms/formats of working together and implement projects in the field (e.g. community-based mechanisms)	Include biological risks and correspondent responses	–
	Prevention measurers (e.g. locally based strategies)	Improve cyber-security on virtual platform	–
	–	To normalize social distancing (e.g. building strategies to keep physical presence with the corresponding preventive measures)	–
Likelihood of CDP continuation	No, unless the whole structure of the CDP changes	–	No, unless the whole structure of the CDP changes
	Yes, although with less presence on the ground	Yes, competition for funds is too high	–
Future ideas for CDP risk-adapted strategies	To build a vision upon risk-adapted strategies that include new categories of unpredictable externalities (e.g. reorganize priorities based on local needs and interests)	To include educational and pedagogical tools in risk-adapted strategies (e.g. preventive measures to prevent violence under unexpected circumstances)	Biosecurity protocol for locals (e.g. healthcare, prevention measures)
	To include negotiation mechanisms to overcome unexpected externalities	–	To establish online platforms to sell local products (e.g. contactless measures)
	To rely less on international expertise (e.g., reducing travels) and more on local capacities	To invest more on environmental and conservation projects (e.g. biodiversity and environmental services as prevention mechanisms to future pandemics)	
CDP evaluation during times of COVID-19 (on a scale from 1, very negative, to 5, very positive)	3.7	4.5	1.9

Source: Authors’ own elaboration

## Discussion

The analysis of CDP responses and risk-adapted strategies offers critical perspectives about peacebuilding. In discussing survey results, we highlight key challenges and coping strategies of implementing agencies and stakeholders. Using peacebuilding theories, we focus on specific world experiences (e.g., COVID-19) and its aspects (Heft 1997). Sample composition—affect by finite time and financial capacity—is the main study limitation. Only a few medium and small CDPs were involved. Additionally, with the continuous development of the pandemic, perceptions are changing rapidly. Thus, the results may be biased. Another possible limitation relates to the degree to which our analysis tends to generalize. For example, most of our data regards local perspectives and sample size is small. Thus, results might not translate or be transferrable to a broader context. A larger sample size across more regions, a more heterogeneous set of stakeholders, and the inclusion of donors’ perspectives could have strengthened this work.

The perspectives and approaches of implementing agencies and stakeholders show similarities and differences. Our findings show universally substantial impacts of COVID-19 on CDPs. While project self-evaluation remains positive before and during the pandemic for large and medium CDPs, small CDPs are negatively affected. Further, the initial perceptions of large and medium CDPs included elements of surprise and scepticism, while small CDPs responded with immediate collective paranoia, especially with respect to the health emergency. Because COVID-19 stands out for its speed of spread, driven by today’s global society, remote rural communities, where small CDPs are implemented, may feel more exposed due to its lack of health services and infrastructure. Historically, poor law-implementation, limited state presence, weak implementations of conflict-prevention measures (e.g. conflict early warning and response mechanisms), high corruption, and inequitable access to services and resources are conditions that make addressing COVID-19 even more challenging (Jayawickreme et al. 2017; Quinn 2016; Rossi et al. 2006). Ultimately, globalization’s negative consequences primarily affect the poor and marginalized in developing countries (Jenkins 2005).

As a result, internal adaptation plans included both the interruption, in some cases preventatively (e.g. small CDPs), and/or the transformation of CDP. Under COVID-19, most projects shifted field activities from physical to virtual. While, some cases demonstrate how CDPs in rural areas can transform and continue, supported by governmental incentives, social cohesion and new conflicts may arise from “digital fatigue”, electricity cuts, and the lack of internet access. Although increased misinformation sharing is a problem rooted in increased internet access, mental disorders are also caused by physical distancing and social/community isolation (Burgess and Fonseca 2020; Islam et al. 2020; Monteith et al. 2020).

Informal measures are re-shaping power relations at the local level with the rise of new and old armed groups contending territorial control (e.g. access entry-exit). Paramilitary and criminal groups are expanding their influence in rural areas, further exploiting natural resources and pursuing illicit activities (e.g. drug production). With the excuse of lockdowns, the consolidation of territorial control increases the vulnerability of civil society and, in particular, those in indigenous communities (Sandoval et al. 2020). Two aspects of trust are emerging: a renewed internal trust in rural community (e.g. solidarity) and a decreasing trust toward institutions and their measures. The latter represent external adaptation components that continue to be enacted. CDPs perceive a top down, central system that delegates functions to regional and local governments, providing funding to assist the most vulnerable communities. In most cases, actions promoted by the central government are perceived negatively, increasing bureaucracy that overlaps with perceived increases in (institutional) corruption, environmental deterioration, food insecurity, human rights violations, and violence against social and environmental leaders.

While large CDPs have greater financial and human capacities to meet such challenges, relocate funds, and implement biosecurity programs, especially in rural contexts, great resilience is also shown by medium and small CDPs, which find creative ways to cope with reduced field activities. Yet, government responses actually increase the complexity of the emergency situation by adding multiple challenges that directly affect the peace, thus hindering direct and indirect protection mechanisms against violence.

Because of lockdown, social alienation might be the main component undermining CDP peacebuilding efforts. Empirical studies show that COVID-19 lockdown measures are affecting mental health and collective trauma (Montoya 2020; Van Bavel et al. 2020); especially problematic for existing post conflict communities. Further, COVID-19 is perceived as negatively affecting Colombian peacebuilding. These challenges and changes in power relations reflect a centralized system unable to support those who are most marginalized, threating CDPs located in rural, remote areas.

Most CDPs are optimistic about the continuation of activities and funds for development and cooperation. Small CDPs are less optimistic, since they face new structural approaches for project implementation. Our findings show that, in times of emergency, learning opportunities arise. First, schemes for territorial planning to foster environmental projects need reconsideration. An *increasing* literature argues that sustainable development is the only way to achieve long lasting peace (Holden et al. 2017; Mansell and Tremblay 2013; Rieckmann 2017). Second, in facing the forthcoming COVID-19 depression, investments in private-public cooperation to sustain small- and medium-scale economic activities is needed, especially in rural areas. Third, strategies for preventing and controlling future pandemics are needed.

Future ideas for CDP risk-adapted strategies include creating new categories of unpredictable externalities (e.g. reorganize priorities based on local needs and interests), educational and pedagogical tools, as well as specific biosecurity protocols for locals. From a local perspective, increasing investments in environmental and conservation projects (e.g. biodiversity and environmental services as prevention mechanisms to future pandemics) is perceived as a key coping strategy that encourages peacebuilding and the implementation of CDPs in times of unexpected externalities.

Finally, the development of project strategies that address risks and uncertainty can be better guided by social learning processes. In this sense, social learning facilitates the integration of different kinds of knowledge (Wals 2007), especially when collectively deciding on actions to cope with crisis situations, unpredictable externalities, or future uncertainty. As proposed by Morin (2002), learning to confront uncertainty is essential knowledge for the future.

## Conclusions

This work highlights key challenges and coping strategies of implementing agencies and stakeholders during the COVID-19 pandemic. The increasing complexity caused by top down governmental measures, effective or not, along with the rise of new local level power relations (e.g. armed groups, illicit activities, environmental deterioration etc.) and social alienation are negatively affecting peacebuilding in Colombia. Remote rural communities (e.g. where small CDPs are implemented) are the most exposed due to the lack of infrastructure (e.g. health services, presence of the state, electricity etc.) and their historic vulnerability (in case they are economically autonomous). Further, the increasing erosion of trust between locals and institutions, along with food insecurity and expected unemployment scenarios due to the COVID-19 depression will not only increase the risk of violence and social unrest, but also the survival of CDPs. In the midst of a crisis, policies and economic efforts should target rural contexts, where peace is most at stake; supporting initiatives historically carried by CDPs such as ceasefires, the mutual assistance and intercommunity cooperation; thus preventing immediate violence and contributing to a construction of more lasting peace in the future. While investments in private-public cooperation and in environmental projects are perceived as key coping strategies against COVID-19, future implications for peace and peacebuilding remain unclear. Thus, further research and monitoring approaches are needed.

## Appendix

**Table 3:** CONTEXT and CDP INFORMATION

	Expertise	Project Role	Project Scale	Project area (departments)	Project duration (years)	Project funds (€ in million)	Project donor (scale)	Project team (*n*.)
*CDP Large*	Peace-process and national affairs	Regional coordinator	Local	Caquetá	1 to 5	>1 M	National	>30
	Peace-process and national affairs	Director	Local	Cesar	>10	>1 M	National	10 to 30
	Peace-process and national affairs	Regional coordinator	Regional	Chocó Cauca Nariño Valle del Cauca	1 to 5	>1 M	International	<5
	National affairs	Director	Local	Caquetá	>5	>1 M	International	10 to 30
	National affairs	Regional coordinator	Regional	(Tumbes-Chocó-Magdalena—Colombia) Chocó Cauca Nariño Valle del Cauca	1 to 5	>1 M	International	>30
	International affairs	Director	Local, regional and national	Caquetá Meta	1 to 5	>1 M	International	10 to 30
	National affairs	Research assistant for monitoring the implementation of the peace agreement	National	Cundinamarca	>10	>1 M	International	10 to 30
*CDP Medium*	Peace-process, national and international affairs	Director	National	Atlántico Cauca Cundinamarca Nariño Valle del Cauca	1 to 5	<1 M	National	5 to 10
	Peace-process	National coordinator	National	Amazonas Antioquia Arauca Atlántico Boyacá Caquetá Cauca Chocó Córdoba Cundinamarca Guajira Guaviare Huila Meta Nariño Putumayo Sucre Vichada	1 to 5	<1 M	International	10 to 30
	Peace-process, national affairs	Regional coordinator	Regional	Caquetá Guaviare Cundinamarca	>5	<1 M	International	1 to 5
*CDP Small*	Peace-process	Regional coordinator	Regional	Cundinamarca Tolima	<1	<1 M	International	>30
	National affairs	Legal representative	Local	Caquetá	>10	<1 M	National	5 to 10
	Local affairs	Officer	Local	Caquetá	>5	<1 M	National	1 to 5
